# The functional and clinical roles of liquid biopsy in patient-derived models

**DOI:** 10.1186/s13045-023-01433-5

**Published:** 2023-04-08

**Authors:** Ziqing Zhu, Erya Hu, Hong Shen, Jun Tan, Shan Zeng

**Affiliations:** 1 Department of Oncology, Xiangya Hospital, Central South University, Changsha, 410008 Hunan People’s Republic of China; 2 Key Laboratory for Molecular Radiation Oncology of Hunan Province, Xiangya Hospital, Central South University, Changsha, 410008 Hunan People’s Republic of China; 3 National Clinical Research Center for Geriatric Disorders, Xiangya Hospital, Central South University, Changsha, 410008 Hunan People’s Republic of China; 4 Department of Neurosurgery, Xiangya Hospital, Central South University, Changsha, 410008 Hunan People’s Republic of China

**Keywords:** Liquid biopsy, Patient-derived xenograft, Patient-derived organoid

## Abstract

The liquid biopsy includes the detection of circulating tumor cells (CTCs) and CTC clusters in blood, as well as the detection of, cell-free DNA (cfDNA)/circulating tumor DNA (ctDNA) and extracellular vesicles (EVs) in the patient's body fluid. Liquid biopsy has important roles in translational research. But its clinical utility is still under investigation. Newly emerged patient-derived xenograft (PDX) and CTC-derived xenograft (CDX) faithfully recapitulate the genetic and morphological features of the donor patients’ tumor and patient-derived organoid (PDO) can mostly mimic tumor growth, tumor microenvironment and its response to drugs. In this review, we describe how the development of these patient-derived models has assisted the studies of CTCs and CTC clusters in terms of tumor biological behavior exploration, genomic analysis, and drug testing, with the help of the latest technology. We then summarize the studies of EVs and cfDNA/ctDNA in PDX and PDO models in early cancer diagnosis, tumor burden monitoring, drug test and response monitoring, and molecular profiling. The challenges faced and future perspectives of research related to liquid biopsy using patient-derived models are also discussed.

## Introduction

**Fig. 1:**
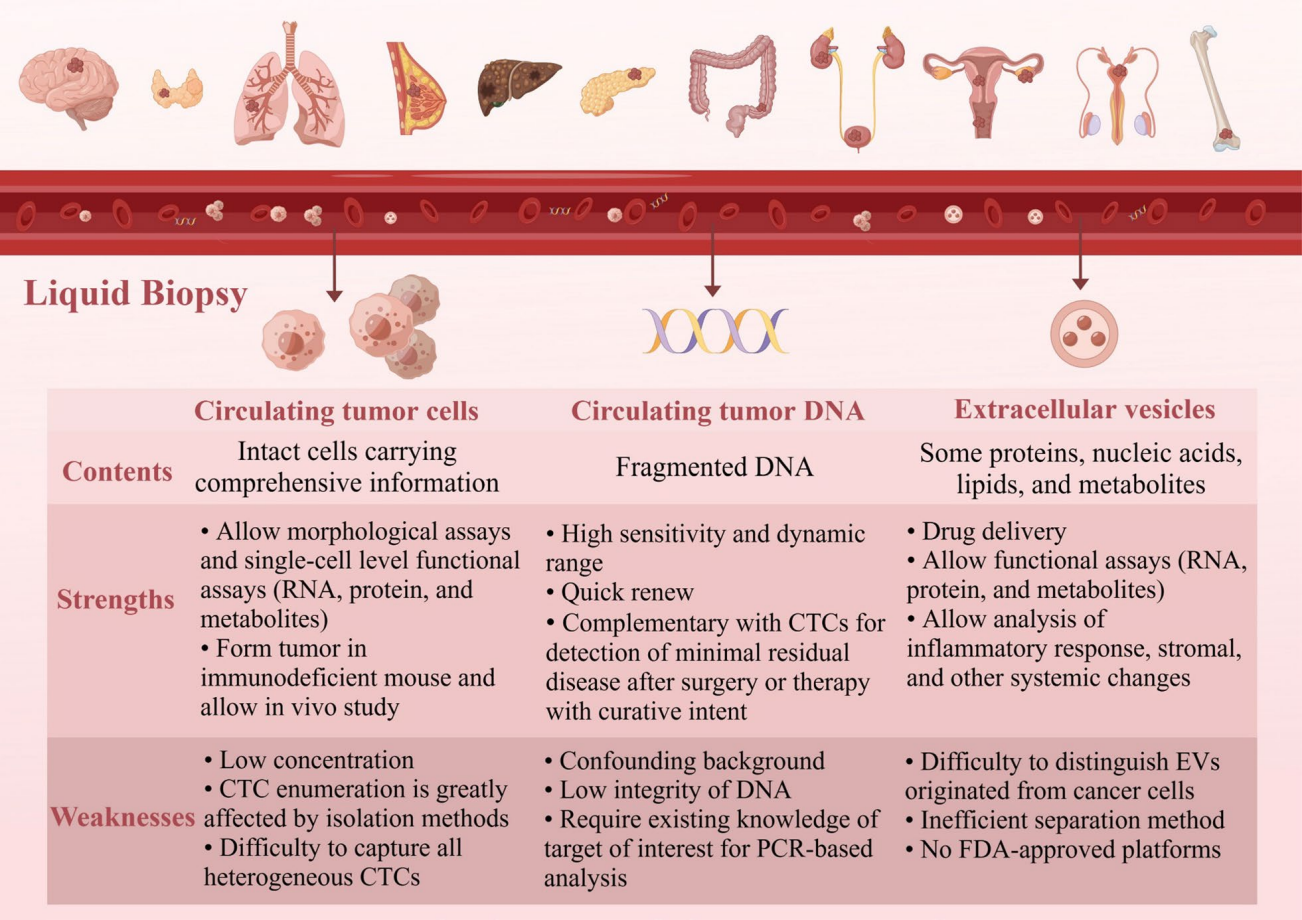
An overview of liquid biopsy. The main contents of liquid biopsy include the analysis of CTCs, EV, and cfDNA/ctDNA, which have their own strengths and weaknesses. CTC: circulating tumor cell; cfDNA: cell-free DNA; ctDNA: circulating tumor DNA; EV: extracellular vesicle. By Figdraw

Liquid biopsy in cancer management has gained attention over the last ten years because of its relative ease of attainment, sequential availability, and minimally invasive procedure compared with traditional tissue biopsy. Any body fluid sample such as blood, urine, saliva, or cerebrospinal fluid can be used in a liquid biopsy; however, blood samples have been the most extensively studied. The most widely studied analytes include circulating tumor cells (CTCs) and CTC clusters, cell-free DNA (cfDNA)/circulating tumor DNA (ctDNA) and extracellular vesicles (EVs) [1] (Fig. 1).

CTC, which is called the “seed” of a tumor, departs from a tumor lesion and enters the peripheral blood circulation and travels to distant organs or tissues in the body, eventually leading to metastases or tumor recurrence [2]. CTCs can also form clusters with other CTCs or other cells such as white blood cells, which are called CTC clusters [3]. CTC detection has many applications in the clinical setting, including prognosis evaluation [4], disease monitoring [5], and treatment personalization [6]. Significantly, CTCs not only predict prognosis as biomarkers but also play an important role in tumor metastasis initiation [2]. Nevertheless, owing to CTCs scarcity and technical hurdles related to the ex vivo culture of CTCs, our understanding of the biology of CTCs and the metastasis mechanism remains in its infancy, making it hard to define potential drug targets and promising biomarkers.

While CTCs carry a complete set of information (including DNA, RNA, proteins, and metabolites, etc.), cfDNA/ctDNA and EVs represent fragmented information about the tumors. CfDNA originating from cell death and active secretion is fragmented double-stranded DNA [7], while tumor-derived cfDNA, also called ctDNA, only makes up a small fraction of it [8]. Point mutations, gene fusions, copy-number variations (CNVs) aneuploidy, epigenetic modifications, and fragmentation patterns are the main tumor-specific alterations [9, 10]. EVs are cell-derived membranous structures, which carrying DNA, RNA, proteins, lipids, oligosaccharides, and metabolites form an independent and stable compartment and prevent contents from enzyme degradation in the extracellular environment [11]. EVs are abundant in body fluids and cancer cells release more exosomes than normal cells to affect the progression, metastasis and treatment of cancer [12]. However, the heterogeneity of cfDNA/ctDNA and EVs in the circulatory system limit their applications as biomarkers. Moreover, cfDNA/ctDNA and EVs are underutilized as markers of drug efficacy due to the inability of high-throughput screening for drugs in humans.

The patient-derived xenograft (PDX) model is established by obtaining tumor tissues from patients through surgery or biopsy and transplanting them into immunodeficient mice through heterotopic (e.g., subcutaneous) engraftment or orthotopic transplantation [13]. Other than tumor tissues, CTCs can also form tumors in immunocompromised mice, called CTC-derived xenograft (CDX) [14]. Patient-derived organoid (PDO) as a 3D construct originates from stem cells, including adult stem cells (AdSCs) and induced pluripotent stem cells (iPSCs) [15]. CTCs can also be used to construct an organoid [16]. PDX and PDO are promising preclinical models, due to their retention of tumor characteristics to the greatest extent. In CTC-related research, PDX and CDX models provide an abundant source of CTCs and serve as systems for mechanistic exploration of CTCs’ metastatic potency, and thus may provide a rationale for biology-driven therapeutics and instructive clinical biomarkers. In addition, the establishment of CDXs from readily accessible peripheral blood collected at different time points during disease progression can overcome some of the restraints of existing models and offers the opportunity to explore acquired drug resistance, and allows disease modeling of the patient who does not undergo tissue biopsy. And for the cfDNA/ctDNA and EVs, the patient-derived models provide a relatively purified source of these analytes and a quick platform to evaluate their clinical potential.

In this paper, we focused on studies using PDX, PDO, CDX, and CTC-derived organoid models for CTCs analyses. We also reviewed the progress of scientific research in studying cfDNA/ctDNA and EVs in patient-derived models. At the same time, we identified essential efforts to make these models more applicable for research on the liquid biopsy analytes so as to expand the clinical applications of the liquid biopsy.

## Main text

## Studying CTCs and CTC clusters in PDX and PDO

PDX faithfully reflects the features of the original tumor and provides a continuous source of CTCs while PDO serves as an alternative model with a lower cost and a higher success rate. There has been increasing interest in using patient-derived models to study human CTCs or CTC clusters captured from mouse blood (Table 1).

**Table 1:** CTC detection in PDX models

Tumor type	Mice	No. of pdx lines	Metastasis	CTC DR	Method	References
Metastatic TNBC	NOD/SCID	2	100%	100%	Flow cytometry	[17]
Metastatic and non-metastatic	NOD/SCID	5	10/18	N/A	N/A	[18]
Metastatic and non-metastatic BC	SCID/beige or NSG	18	50	83%	Anti-human pan-CK IHC	[19]
NA	SCID	7	0	2/7	EpCAM-based platform	[20]
Metastatic TNBC	NOD/SCID	3	70	11/72	FACS-based assay	[21]
BC	SCID	1	NA	2/7	RT-qPCR	[22]
TNBC	NSG	7	23/57	32/37	Microfluidic chip	[23]
TNBC	NSG	3	13/17	22/23	Label-free platform based on physical characteristics	[24]

N/A, not available; NOD, non-obese diabetic; SCID, severe combined immunodeficient; IHC, immunohistochemistry; NSG, NOD scid gamma; DR, detection rate; qRT-PCR, quantitative real time polymerase chain reaction; EpCAM, epithelial cell adhesion molecule; BC, breast cancer; FACS, fluorescence-activated cell sorting; TNBC, triple-negative breast cancer; CRC, colorectal cancer

### Circulating tumor cell

The CTC shedding process was studied in PDXs. E. Powell and colleagues developed paired triple-negative breast cancer (TNBC) PDX models with the only difference being *p53* status. They reported that CTC shedding was found to be more related to total primary and metastatic tumor burden than *p53* status [17]. Research on larger metastases, which was made possible by tumor excision surgery, revealed that mesenchymal marker vimentin-expressing CTCs and the presence of CTCs clusters were associated with a greater distribution of metastatic burden in the lung and liver [24]. It is notable that while CTCs can be detected in a PDX line of five tumor-bearing mice, no distant metastatic lesions were detected in them [25]. The single-cell RNA sequencing of primary tumor cells, liver metastatic cells, and CTCs from a highly metastatic pancreatic ductal adenocarcinoma (PDAC) PDX demonstrated decreased expression of cell cycle and extracellular matrix-associated genes in CTCs. One of the most highly upregulated genes in CTCs was *Survivin* (*BIRC5*), a key mediator of mitosis and apoptosis. Treatment with an inhibitor of survivin YM155 alone or in combination with chemotherapy hindered metastatic development and led to improved survival of metastatic PDX models [26]. Since it is hard to culture CTCs outside of their natural environment, PDXs are great platforms to study the functional roles of CTCs and the important pathways that support those roles. After defining targetable pathways related to CTC shedding, survival and extravasation, PDXs can then be used to test the novel targeted therapy.

Tumor cells can undergo epithelial-mesenchymal transition (EMT), a process that initiates the loss of cell–cell adhesion and cell-substrate attachment and promotes CTC shedding [4]. A. Tachtsidis et al. analyzed changes in 41 epithelial‐mesenchymal-plasticity (EMP)-related genes and found a largely agreeable pattern of transcript alteration between the cell-line-derived models and the patient-derived models. Intriguingly, a mixed picture was depicted in respect of EMP and metastasis in that although a number of mesenchymal markers were found to be upregulated in CTCs compared to primary tumors, epithelial markers such as *SERPINE1* and *NRP1* were also noted to have significantly elevated expressions [22]. Therefore, it remains a future challenge to determine the intriguing relationship between the CTC’s mesenchymal status and its metastatic potential.

### Circulating tumor cell cluster

CTC clusters are cell clusters composed of two or more tumor cells, mainly derived from primary tumors, metastatic sites, or the aggregation and proliferation of single CTCs [27]. CTC clusters have higher metastatic potential than a single CTC [19]. This was also confirmed in the study of a colorectal cancer (CRC) PDX line [28].

The formation and interaction within a certain CTC cluster is a hot topic. Using TNBC PDX mice that generated spontaneous lung micrometastasis, Xia Liu et al. unveiled a novel mechanism of human TNBC CTC cluster formation via CD44/PAK2-mediated cellular aggregation [29]. This finding adds to the previously proposed theory of collective migration and cohesive shedding of polyclonal CTC clusters [30–32]. In the clinical setting, the presence of CD44-positive CTC clusters correlates with poor overall survival (OS), highlighting its potential role as a prognostic marker and a therapeutic target. Afterwards, an ex vivo 3D culture system of TNBC PDX primary tumor cells identified clone LA1, an epidermal growth factor receptor (EGFR) monoclonal antibody as a CD44 blockade antibody and the tumor suppressor microRNA-30c as effective inhibitors of circulating cancer stem cell (CSC) cluster formation in vitro and lung metastasis of TNBC in vivo [33]. Their further study combined single-cell RNA sequencing and protein analyses of the paired primary tumor and lung metastatic lesion from TNBC PDX mice. They reported ICAM1 as a key metastatic initiator through homophilic ICAM1-ICAM1 interactions, enhancing homotypic CTC cluster formation. The inhibition of ICAM1 drastically blocked tumor cell cluster formation, transendothelial migration, and lung colonization [34]. These studies using PDX models held great promise for extending CTC's potential role in diagnostic prediction and helped the development of therapeutics that prevent and block polyclonal metastasis.

Due to the rarity of the CTC clusters, it is rather important to identify the molecular characterization of the primary tumors that bring about CTCs. By analyzing reverse-phase protein array (RPPA) and transcriptomic (RNA-Seq) data of six CTC cluster-negative and four CTC cluster-positive (CTCcl+) TNBC PDX models, the author found relatively elevated expression of Bcl2 and reduced expression of *ACC1* in CTCcl+ models [35]. Moreover, the gene signature predicting the presence of CTCcls correlated with worse relapse-free survival in a publicly available dataset of 360 patients with basal-like breast cancer (BC) [35]. These molecular patterns that are related to CTC clustering may become applicable clinical predictive biomarkers in the future.

**Fig. 2:**
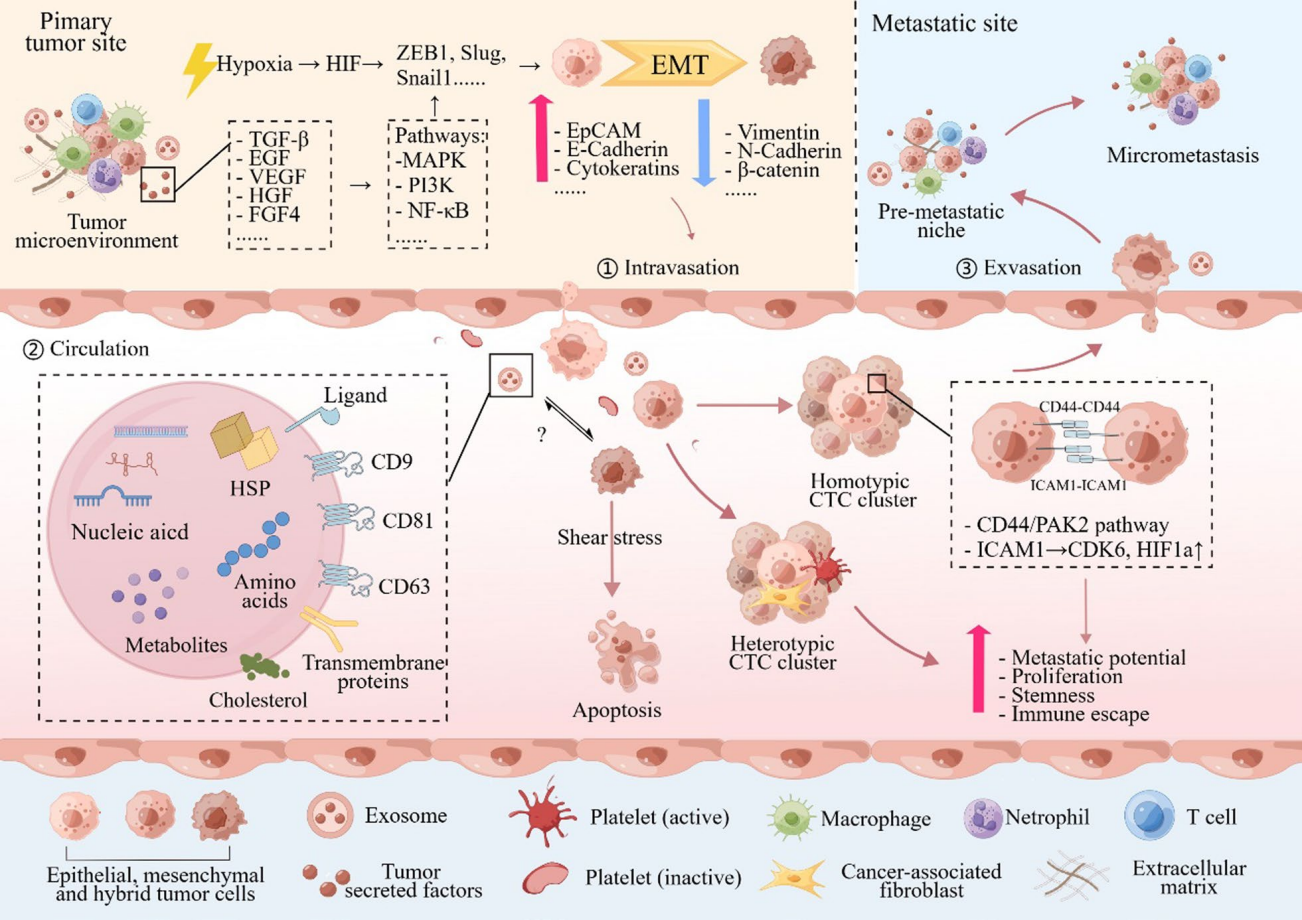
Key stages of metastasis relating to CTC generation and colonization. In the primary tumor site, tumor cells undergo EMT triggered by extracellular molecules and tumor microenvironment stimuli such as hypoxia. EMT promotes CTC shedding and intravasation. In circulation, only a small proportion of CTCs can survive shear stress. CTCs can form CTC clusters with each other or with other immune cells or cancer-associated fibroblasts, which enhances their metastatic potential, proliferation ability, stemness, and immune escape. Eventually, tumor cells form a pre-metastatic niche and colonization at a distant site after CTC extravasation. EVs also play an important role in these stages, mediating cell-to-cell communication and shaping the pre-metastatic niche. However, more research is needed to understand the interaction between EVs and CTC. CTC: circulating tumor cells; EMT: epithelial-mesenchymal transition; EVs: extracellular vesicles; HSP: heat shock protein. By Figdraw

The key metastatic stages related to CTCs shedding and colonization are shown in Fig. 2. Using PDX models, studies on CTC can shed light on the biological process of CTC shedding, studies on CTC clusters can uncover the important mechanism behind CTC cluster formation and its enhanced metastatic potential, thus finding novel treatment targets to prevent cancer metastasis. More importantly, tumor heterogeneity and genetic studies were ensured in animal models since PDX models are constructed by direct implantation of patients' own tumor tissues. Further explorations are required to compare the features of liquid biopsy materials within various immunocompromised models and with their humanized components in the human immune system. We also expect to see studies of liquid biopsy analytes in humanized mice with human immunity or in complex co-culture organoid models with cancer-associated fibroblasts or immune cells.

### CDXs and CTC-derived organoids in cancer research

#### The generation of CDXs and ex vivo culture of CDX cells

The first successful CDX models were generated in 2013 by injecting a BC CTC-derived cell line intracardially or into the tail vein of immunodeficient mice [36]. Later studies proved the tumor-initiating potential of CTCs. Elisabetta Rossi and her team subcutaneously injected CTCs from breast or prostate cancer (PCa) patients into NOD/SCID mice. At 6.5–12 months after CTC injection, a single CTC could be detected in the peripheral blood, bone marrow, and spleen, though no mice had developed tumors [37]. One year later, Cassandra L. Hodgkinson and colleagues successfully generated 4 CDXs from small cell lung cancer (SCLC) patients. Further research showed that CDXs faithfully maintained the original tumor's genetic and morphological characteristics and reflected the patients' responses to chemotherapy [38]. CDXs have been shown to be reliable models for disease modeling and drug testing [38]. They also make it possible for the generation of matching animal models when tissue biopsies are rarely done in certain kinds of cancers such as SCLC.

The development of CTC isolation and enrichment strategy contributed to the efficiency of CDX generation. Researchers applied a microfluid-based device to CTC enrichment and produced CDXs with an efficiency of 38%. Notably, they collected blood samples at relevant timepoints along the same patient's disease course and generated serial CDXs. This series of CDXs enabled future investigations of acquired chemoresistance and offered a novel chemo-resistant model for drug testing [39]. The CTC capture and isolation technologies are still evolving; hence we can expect more efficient CDX construction process and a wider application in the clinical setting.

Till now, CDX models have been successfully generated from patients with SCLC [38], non-small cell lung cancer (NSCLC) [40], BC [41], liver cancer [42], melanoma [43] and PCa [44] (Table 2). For highly efficient CDX generation, factors that may influence CDX generation should be determined. CTC counts presumably affected the success rate [38, 45]. For example, the Dive Lab reported two successful CDX generations out of 146 attempts from samples with a CTC count < 49, in contrast to 35 successful CDX generations out of 71 efforts from samples with a CTC count > 50 [46]. Clinical data from a report found significantly shorter progression-free survival and overall survival (OS), poorer baseline performance status, an increased chance of having chemo-refractory disease, and a higher proportion of liver and bone metastases in SCLC patients whose samples effectively generated CDX. Interestingly, the stage at diagnosis does not make a difference, which may be attributed to the existence of prevalent micrometastases at diagnosis [47].

**Table 2:** CTC-derived xenograft models established to date

Tumor type	Stage	CTC counts (/7.5 ml) and enumeration method	Enrichment and characterization	Injection procedure	Mice	Number	Success rate	Passage	References
BC	Metastatic luminal	> 1109 EpCAM + (CellSearch)	Purified by FACS (PI-CD45-EpCAM +) after depletion of hematopoietic cells using the RosetteSep kit	Injection in femoral medullar cavity	NSG	6 CDXs	5%	N/A	[45]
	Metastatic triple-negative	969 CTCs EpCAM + (CellSearch)	Density gradient centrifugation: Histopaque®	Subcutaneous injection/orthotopic injection	NUDE/Scid Beige	1 CDX line	3%	Piece of tumor explant or injection of explant culture	[41]
	Triple-negative	N/A	By FACS (CD45/CD34/CD105/CD90/CD73 −)	Intracardiac injection	NSG	1 CDX line	N/A	Minced metastatic liver tissue	[48]
	Metastatic luminal	N/A	Parsortix	Orthotopic injection	NSG	1 CDX	N/A	N/A	[49]
SCLC	Metastatic	> 400 EpCAM + (CellSearch)	RosetteSep	Subcutaneous injection	NSG	4 CDX lines	67%	Tumor fragments	[38]
	N/A	N/A	CTC-iChip or RosetteSep Ficoll	Subcutaneous injection	NSG	5 CDX lines	38%	Tumor fragments	[39]
	N/A	0–49 (67%) > 49 (33%) EpCAM + (CellSearch)	RosetteSep	Subcutaneous injection	NSG	38 CDX lines	17%	Tumor fragments	[46]
	Extensive-stage	N/A	RosetteSep	Subcutaneous injection	NSG	8 CDX lines	N/A	Tumor fragments	[50]
NSCLC	N/A	> 150 CD45/CD144/vimentin/CK (FACS)	RosetteSep	Subcutaneous injection	NSG	1 CDX line	100%	Disaggregation of tumor	[40]
	Non-metastatic	N/A	RosetteSep	Subcutaneous injection	NSG	2 CDXs	20%	N/A	[51]
	Advanced	693 on average with a median of 9 EpCAM + (CellSearch)	RosetteSep	Subcutaneous injection	NSG	3 CDXs	7.3%	Tumor fragments	[52]
LUAD	Stage IIa	CTC-TJH-01 cell line	Microfluidic Herringbone-Chip and immunomagnetic microbeads	Tail vein injection	NOD/SCID	1 CDX	N/A	N/A	[53]
Melanoma	Stage III or stage IV	N/A	By multi-parametric flow sorting (CD45-/CD34-/CD90-/CD73-/CD105-cells) and validation by FACS (S100 and Melan-A)	Injection into the left ventricle	NSG	8 CDXs	100%	N/A	[43]
	Stage IV	N/A	RosetteSep	Subcutaneous injection	NSG	7 CDXs	15%	N/A	[54]
HCC	Advanced	N/A	Purified by FACS (EpCAM + /CD45 −) after depletion of PBMCs	Subcutaneous injection	NOD/SCID	3 CDXs	50%	N/A	[42]
Colon Cancer	N/A	CTC-MCC-41 cell line	RosetteSep	Subcutaneous injection	SCID	1 CDX	N/A	N/A	[55]
PCa	Metastatic	N/A	By FACS (CD45-) after depletion of PBMCs	Subcutaneous injection	NSG	N/A	N/A	N/A	[44]
	Metastatic castration resistant	19,988 CTCs xenografted in total EpCAM + (CellSearch)	DLA + RosetteSep	Subcutaneous injection	NSG	1 CDX line	14%	Tumor fragments	[56]
BC and PCa	Metastatic	50 to 3000 EpCAM+ (CellSearch)	CellSearch (EpCAM+)	Subcutaneous injection	NOD/SCID	8 CDXs	100%	N/A	[37]

BC, breast cancer; EpCAM, epithelial cell adhesion molecule; NOD, non-obese diabetic; SCID, severe combined immunodeficient; NSG, NOD scid gamma; CTC, circulating tumor cell; CDX, CTC-derived xenograft; N/A, not available; SCLC, small cell lung cancer; NSCLC, non-small cell lung cancer; PBMC, peripheral blood mononuclear cell; HCC, hepatocellular carcinoma; PCa, prostate cancer

Considering the expense and duration of CDX model generation, researchers investigated the short‐term ex vivo cultures of CDX cells for drug screening and the biology exploration of SCLC. Their results confirmed ex vivo CDX cells as a novel drug-testing platform with the maintained neuroendocrine (NE) phenotype [57] and non-NE phenotype [58]. Moreover, combined with detailed patient information, ex vivo CDX cells have the advantage of being manipulated for functional analysis, helping to identify new drug targets and metastasis mechanisms.

#### The applications of CDXs in genetic characterization and drug testing

CDX models can offer a platform for the tracking of metastatic disease through genetic characterization and analysis of exclusive mutations in CDXs (but not in the primary tumor biopsy), as well as the testing of biology-driven therapeutic hypotheses. Faugeroux V. and his colleague presented a comprehensive genomic characterization of the primary tumor, CTCs, a prostate CDX of the castration-resistant prostate cancer (CRPC)-NE phenotype, and CDX-derived cell lines. This genomic analysis demonstrates the sequence of gaining the key driver genes (i.e., *TP53, PTEN,* and *RB1*) that direct transformation into CRPC-NE and suggests that this process requires tumorigenic CTCs with CRPC-NE characteristics [56]. Tala et al. focused on DNA damage response (DDR) and genome integrity-related genes characterized in NSCLC CDXs. Key DDR-related mutations emerge from whole-exome sequencing analysis, including *TP53*, *BRCA2*, *CHEK2*, and *ARID1B*, which might associate with CTC-mediated metastatic progression [52]. NGS of the SCLC CTC samples and CDX models observed more frequent copy number aberration (CNA) gains in chemorefractory CDXs and CTCs [38]. A later study managed to build a CNA-based classifier to distinguish chemosensitive SCLC patients from chemorefractory patients according to CNAs in CTCs from the patient, which was validated in six SCLC CDXs [59].

The latest CRISPR screen technology was also employed to genetically characterize BC CDX and define key genes in CTC shedding and metastasis. They injected BC CTCs transduced with Cas9-GFP and sgRNA library into the NSG mice. By identifying sgRNAs present in the primary tumor but not in CTCs, they found that *PLK1* is required for the intravasation of CTCs. They then constructed a “metastasis signature” consisting of 114 genes, which can serve as a predictive tool for metastasis-free survival (MFS) [49]. In another study, the researchers exclusively injected CDX‐derived metastatic liver tissue intracardially in mice and sequentially established four passages to accumulate liver metastatic markers and defined a TNBC liver metastasis gene signature with 597 specific genes [48]. Genetic characterization conducted in CDXs helps uncover driver genes in cancer and potential metastasis-related gene patterns which can then be used in clinical practice.

CDX models carried tumors formed by CTCs and represent minimal residual disease or micrometastasis targeted by adjuvant chemotherapy, whereas PDX models represent the primary tumor [49], which makes CDX a better preclinical model for chemo drug testing. The SCLC CDX was used to test the efficacy of GDC-0941, a *PI3K* inhibitor, and Navitoclax, a Bcl-2 inhibitor, alone and in combination [60]. It is noteworthy that in a study, researchers treated mice bearing CDX with a single cycle of cisplatin/etoposide to induce an initial tumor response. In this way, for the first time, they could study acquired treatment resistance in SCLC CDX models, providing new test-beds to test drugs in both first-line and second-line settings [61]. The biggest advantage of CDXs is that they ensure sequential modeling along the disease course. With the help of the latest multi-omics tools, CDXs can provide answers to the underlying mechanisms behind metastasis and acquired resistance to chemotherapy, unveiling relevant therapeutic targets. Besides, CDX possesses great potential for testing candidate therapies and supports the growing body of evidence for tailor-made therapies based on the molecular stratification of tumors. For example, a CDX can be generated from a patient enrolled in a clinical trial and treated with the same experimental regimen to recapitulate the clinical response, called a co-clinical trial. A CDX model can be a perfect co-clinical trial model since it only needs blood samples from the patient and can be established before and after the treatment, providing vital information for disease progression, drug resistance mechanism, and predictive biomarkers.

#### CDX allows the decoding of tumor heterogeneity

Clonal homogeneity was once considered a distinct feature of SCLC. NGS analysis reported the presence of a concordant somatic TP53 mutation in all SCLC CTCs examined and a high degree of overall resemblance in the CNA patterns of SCLC CTCs and CDX [38]. In another study, the researchers found retention of somatic alterations in matched SCLC primary tumors, and initial model generation (P0) and serial passages of PDXs and CDXs, indicating relative genomic fidelity and homogeneity [39]. However, later studies challenged this notion.

The Dive Lab explored inter- and intratumoral phenotypic heterogeneity in a large biobank of 38 SCLC CDX models from 31 patients, including six longitudinal CDX pairs generated at baseline and post-relapse. Intertumoral heterogeneity is evident at the level of *ASCL1*, *NEUROD1*, *POU2F3*, and MYC family genes (*MYC, MYCL, MYCN*). Within *ASCL1* + /*NEUROD1* + CDX, intratumoral heterogeneity was well-defined with mutually exclusive parts of *ASCL1* and *NEUROD1* positive cells [46]. The Dive lab portrayed another level of intratumoral heterogeneity by finding that a rare section of CTCs presents a vascular mimicry (VM) phenotype with VE-cadherin expression and defining PAS + /CD31 − VM vessels in SCLC CDX models [62]. They also found a rare YAP1 subtype of SCLC in CDXs [63]. Together, they managed to define the four SCLC subtypes on the basis of transcription factor expression of ASCL1, NEUROD1, POU2F3, and YAP1, in CDX models [64, 65].

CDXs also assisted in elucidating tumor heterogeneity at the single-cell level. One study performed single-cell analysis on four platinum-sensitive and four platinum-resistant SCLC CDX models. CTC and CDX cells analyses revealed lower profiles of *MYCL* and *NFIB* in CDX tumors, which were probably expressed by circulating metastatic cells but were not required in the primary lesion. They also calculated an intra-tumor heterogeneity (ITH) score to quantify heterogeneity and predict chemo response with higher ITH scores in the platinum-resistant CDXs. Nonetheless, it is hard to depict the characteristics of platinum-resistant CDXs because substantial diversity was found between clusters of a single CDX, which requires further investigation. tSNE clustering showed that the vehicle-treated cell cluster is completely separated from the cisplatin-relapsed cell cluster, with a greater ITH score in the cisplatin-treated tumor in comparison to the vehicle-treated tumor. It is noteworthy that the onset of platinum resistance was linked to a loss of ASCL1-expressing cells with no difference in NEUROD1 expression [50].

To elucidate heterogeneity in NSCLC and CDX tumors, one study used the PDX model for CTC enrichment and subsequent CDX generation to provide enough samples for single-cell analysis. The researchers re-implanted CTCs of primary tumor PDX mice into immunodeficient mice to establish NSCLC CDX models. The single-cell analysis discovered an additional AT2-like population in CDX tumors, validated by external single-cell sequencing data of human patients' NSCLC metastases [51]. Single-cell analysis can reveal detailed information about the tumor microenvironment. CDX models provide abundant, attainable, and renewable samples for single-cell analysis.

#### CTC-derived organoids in cancer research

In 2014, Dong Gao et al. first reported success in constructing an organoid line from the CTCs of a CRPC patient with > 100 CTCs per 8 mL. They showed organoids' suitability for drug testing by testing enzalutamide and two PI3-kinase pathway inhibitors [16]. Different kinds of CTC-derived organoids have been established (Table 3). However, the low median CTC count in patients with metastatic PCa hampered efficient organoid generation [66]. Lisanne Mout et al. innovatively performed diagnostic leukapheresis (DLA) in patients with metastatic PCa for CTC enrichment and successfully established CTC-derived organoids in 14 out of 40 DLA samples and one stable organoid cell line [67]. For early-stage patients with a relatively low CTC count in the blood, Zhang et al. established a three-dimensional (3D) co-culture model to ex vivo expand CTCs from patients with early-stage lung cancer [68].

**Table 3:** CTC-organoid established to date

Tumor type	Stage	CTC counts	Enrichment and characterization	Culture condition	Number	Success rate (%)	References
PCa	Castration-resistant	> 100/8 ml	RosetteSep	Advanced DMEM/F12	1	N/A	[16]
	Metastatic	> = 5/7.5 ml EpCAM+ (CellSearch)	DLA + RosetteSep + CellSearch/DLA + RosetteSep	Adjusted prostate cancer organoid medium (APCOM)	14	35	[67]
Lung cancer	Early-stage	1–11/1 ml CK7/8 (confocal)	A microfluidic CTC-capture device	With fibroblasts extracellular matrix on a microfluidic chip	14	73	[68]
SCLC	Limited-stage or extensive-stage	8–433/7.5 ml IsoFlux	RosetteSep	In DMEM/F12 medium on binary colloidal crystals	18	81.8	[69]
HNC	Stage I–IV	N/A	RosetteSep	Seeded onto a binary colloidal crystal (BCC) substrate and cultured in DMEM/F12 medium	37	92.50	[70]
PDAC	Stage II–IV	N/A	RosetteSep	Seeded onto a binary colloidal crystal (BCC) substrate and cultured in DMEM/F12 medium	36	87.8	[71]

N/A, not available; PCa, prostate cancer; CTC, circulating tumor cell; DLA, diagnostic leukapheresis; SCLC, small cell lung cancer; EpCAM, epithelial cell adhesion molecule; DMEM, Dulbecco's modified eagle medium; HNC, head and neck cancer; PDAC, pancreatic ductal adenocarcinoma

More reports were given by a team from Taipei who successfully achieved ex vivo expansion and drug sensitivity screening of CTCs from SCLC [69], head and neck cancer (HNC) [70], and PDAC [71] patients, utilizing a lab-developed CTC culture platform called eSelect. Instead of using an extracellular matrix, the eSelect platform applied a biomimetic substrate called binary colloidal crystal (BCC) monolayers to construct a complex topographic surface [72]. The CTC-derived organoid culture efficiency for SCLC, HNC, and PDAC has reached 81.8% (18/22), 92.50% (37/42), and 87.8% (36/41), respectively. Also, the team tested novel drugs [73] or commonly subscribed chemotherapies [70] on the models. Together, these results proved that drug sensitivity tests on CTC-derived organoids could predict treatment outcomes in matching patients or at least negate the need for a money and time-consuming procedure of trial-and-error to rule out ineffective treatment regimens.

**Fig. 3:**
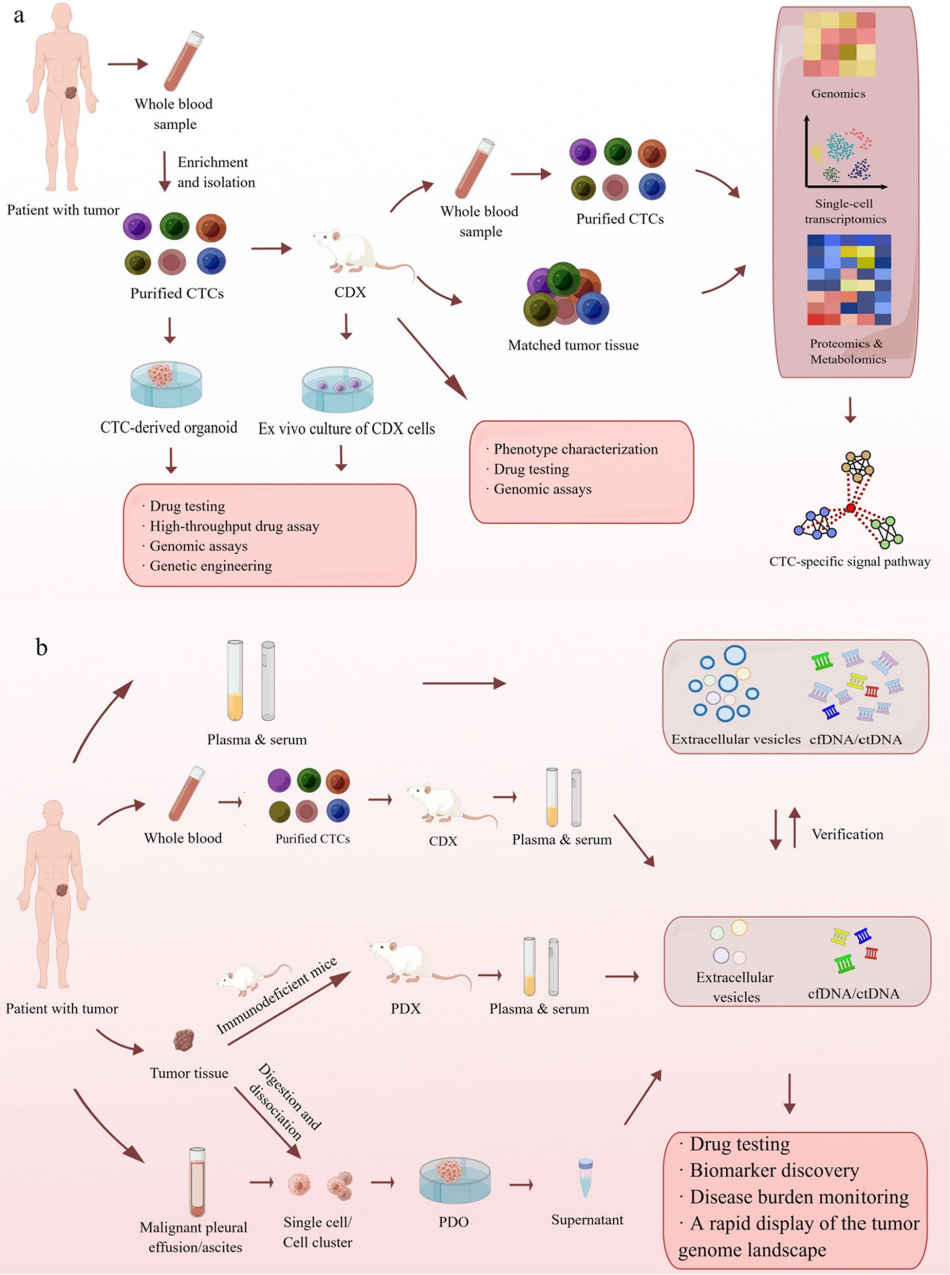
Liquid Biopsy in Patient-Derived Models. **a** CTCs enriched and isolated from blood samples of cancer patients are injected subcutaneously or orthotopically into immunocompromised mice to develop CDXs. Phenotype characterization can confirm the CDXs' fidelity of the primary tumor. CDXs can also be used to test novel drugs and portray a comprehensive tumor genetic landscape. By collecting the blood sample and matched tumor tissue and purifying them into CTCs, CTC-specific signal pathways can be found with genomics, single-cell transcriptomics, proteomics, and metabolomics analysis. Ex vivo cultures of CDX cells can be utilized for drug screening and genome-wide analysis. CDX-derived organoids also need to undergo the phenotype characterization process so that they can be used as a drug testing and high-throughput screening platform and as genomic analysis materials. CDX-derived organoids and CDX cells are amenable to gene editing. **b** Extracellular vesicles and cfDNA/ctDNA can be isolated and analyzed in the plasma and serum from the PDX, CDX, and PDO models, which can apply to drug testing, biomarker discovery, and disease burden monitoring. The tumor-specific variants can be distinguished from the patient’s plasma and serum samples. What’s more, the patient-derived models provide a rapid platform to display the tumor genome landscape. CTC: circulating tumor cell; CDX: CTC-derived xenograft; PDX: patient-derived xenograft; cfDNA: cell-free DNA; ctDNA: circulating tumor DNA. By Figdraw

CTC-derived organoids have the advantages of a higher success rate, shorter generation time, and greater amenability to gene editing compared to CDX. Nevertheless, CTC-derived organoids have only been built in a few cancers, including PCa [16], lung cancer [68], head and neck cancer [70], and PDAC [71]. Additional efforts should be made to establish organoids derived from CTCs of other types of cancer, and to generate a biobank of CTC-derived organoids. Future research can also apply the latest technologies, including high-throughput drug screening, 3D printing, organoids-on-a-chip, and genome editing, to CTC-derived organoids. The applications of CDX and CTC-derived organoid are summarized in Fig. 3a.

### Expanding liquid biopsies of cfDNA/ctDNA and EVs in patient-derived models

#### CfDNA/ctDNA

The potential clinical applications of cfDNA/ctDNA have been explored and put into practice [74]. The sequencing and fragmentation pattern analysis [10] of ctDNA makes it a powerful biomarker for tumor screening, treatment response drug resistance, molecular residual disease, prognosis, relapse and tumor genomic evolution [75]. Blood sampling from patients are more reliable than PDX and PDO models for cfDNA/ctDNA. But there are problems with ctDNA variants’ analysis and background mutation filtering. The analyses of cfDNA mutations requires filtering off background mutations and looking for tumor-specific mutations. The pattern of ctDNA fragments from human and rat sources shows difference [76], making it easier to distinguish ctDNA in PDX models. Wang et al. showed that by taking peripheral blood mononuclear cell (PBMC) as background mutations, alleles with different low frequencies in cfDNA but higher frequencies in primary tumor and PDX models were more likely to be tumor-specific mutations [77]. Further research is still needed to detect and analyze the tumor-specific variants of ctDNA.

The use of ctDNA for tumor burden monitoring has advantages over direct tumor weighing or imaging. For one, the liquid biopsy is a non-invasive monitoring method, that enables sensitive and continuous measurement. For another, the disease burden is related to not only the physical size of the tumor but also the proliferation ability and invasiveness of the tumor cells [78]. Studies have proved that the cfDNA/ctDNA levels in PDX model plasma were able to reflect the tumor burden represented by the tumor volume in different kinds of cancers. [79, 80] In the Cutaneous T-Cell Lymphoma (CTCL) PDX model developed by Wu et al., the level of human plasma β-actin cfDNA correlated with tumor burden [81]. Importantly, β-actin gene is relatively conserved in many kinds of cancer, so it’s a promising target for monitoring [81].

The result of the cfDNA/ctDNA sequencing is supposed to guide molecular-targeted drug use, but the overall response rate seems to be limited, because it remains elusive to distinguish tumor driver variants from a large number of variants. PDX models provide a more direct and efficient way to test the potential drug targets revealed by cfDNA/ctDNA sequencing results and observe the treatment effect [82]. For example, the relapse of CRC is accompanied by MET amplification in ctDNA, and in the PDX model, MET inhibitors weaken the resistance to anti-EGFR therapy brought by MET amplification [83]. PDO models are also applied for the high-throughput drug screening. In the early 72 h after fine-needle aspiration PDOs, genetic mutations detected in the PDO supernatant had a similar profile to the primary tumor. PDOs provide an efficient platform to evaluate the efficacy of drugs, especially for patients treated with neoadjuvant therapy [84]. Analyzing cfDNA/ctDNA in the PDX and PDO models helps with the discovery of biomarkers and tumor burden monitoring (Table 4) (Fig. 3b). Importantly, the results of ctDNA studies in patients and derived models can be mutually explained and verified. Especially, the patient-derived models can complement the analysis of ctDNA from human blood samples [54].

**Table 4:** Analyzing cfDNA and ctDNA in PDX and PDO models

Tumor type	Model	Mice	Sample	Isolation	Analysis	Application	References
GB	PDX	rnu/rnu athymic nude rats	Plasma, cerebrospinal fluid and urine	QIAamp circulating nucleic acids kit	dPCR Shallow WGS	Early screening and diagnosis	[76]
Ewing sarcoma	PDX	NSG mice	Plasma	Qiagen circulating nucleic acid kit	ddPCR	Early screening and diagnosis	[80]
CTCL	PDX	NSG mice	Plasma	NucleoSpin plasma kit	qPCR	Drug testing	[79]
CTCL	PDX	NSG mice	Plasma	NucleoSpin Plasma XS kit	qPCR	Drug testing; Tumor burden monitor	[81]
pRCC	PDX	RAG2−/−γC−/−mice	Plasma	QIAamp DSP virus spin kit	qPCR	Drug testing	[85]
HGSC	PDX	NOD mice	Plasma	Qiagen investigator kit	Shallow WGS	Tumor burden and response monitor	[86]
Pancreatic cancer	PDO	N/A	Supernatant	QIAamp ultrasens virus kit	ddPCR NGS	Molecular profiling and drug testing	[84]
BC	PDX	SCID/Beige mice	Plasma and serum	Quick-cfDNA Serum & Plasma Kit	qRT-PCR	Novel PDX model evaluation	[87]

CfDNA, cell free DNA; ctDNA, circulating tumor DNA; GB, glioblastoma; CTCL, cutaneous T-cell lymphoma; pRCC, papillary renal cell carcinoma; HGSC, high-grade serous ovarian cancer; BC, breast cancer; NOD, non-obese diabetic; NSG, NOD scid gamma; SCID, severe combined immunodeficient; N/A, not available; PCR, polymerase chain reaction; dPCR, digital PCR; ddPCR, droplet digital PCR; qPCR, quantitative PCR; qRT-PCR, quantitative real time PCR; WGS, whole genome sequencing; NGS, next-generation sequencing

#### EVs

EVs have been emerging as a promising biomarker due to their relative abundance, instinct stability, and role in orchestrating cancer metastasis. Patient-derived models show their merits in distinguishing the origin of EVs from tumor cells, tumor stromal cells, or non-tumor tissues. Further, these models can be used to differentiate EVs originating from primary and metastatic sites or from simultaneous primary sites. PDX models also render a chance to specifically enrich EVs released by human cancer cells in the mouse plasma background. Hong et al. developed an acute myeloid leukemia (AML) PDX model and found that the molecular profiles of enriched EVs were similar in the PDX model and patient [88].

PDX and PDO models have been used for biomarker development based on EVs and contents (Fig. 3b). For example, miR-92a-3p is rich in plasma of the HCC PDX model and was shown to promote hepatocellular cancer carcinoma (HCC) metastasis [89]. The clinical data verified that miR-92a-3p can be a biomarker of poor prognosis in HCC patients [89]. With patient-derived models, EVs and their contents have also been proven to predict and monitor cancer occurrence, progression, and prognosis in different kinds of tumors (Table 5).

**Table 5:** Analyzing EV and its contents in PDX and PDO models

Tumor type	Model	Mice	Sample	Subjects	Isolation	Analysis	Application	References
HCC	PDX	Nude mice	Plasma	EV and miRNA	TRIzol reagent for total RNA	HiSeq Rapid SR Cluster Kit V2; qRT-PCR	Diagnositic biomarker	[89]
SCLC	CDX	NSG mice	Plasma	miRNA	miRNeasy kit	qRT-PCR; Human TaqMan Low Density Arrays	Tumor burden monitoring	[90]
AML	PDX	NSG mice	Plasma	Exosome	Mini size-exclusion chromatography	Hunable resistive pulse sensing; Pierce BCA protein assay kit; Transmission electron microscopy	Mechanism of immune suppression	[88]
BC	PDX	NOD/SCID and NSG mice	Plasma	miRNA	Exoquick reagent	qRT-PCR	Potential screening biomarkers	[91]
DLBCL	PDX	NSG mice	Serum	miRNA	QIAgen miRNeasy Mini Kit	cDNA generation and ddPCR	Tumor classification	[92]
PDAC	PDX	NOD-SCID mice	Serum	EV	Centrifugation	Far-field nanoplasmon-enhanced scattering assay	Tumor burden monitoring	[93]
AML	PDX	NSG mice	Plasma	EV	Ultracentrifugation	High-resolution microscopy NGS for miRNA	Tumor burden monitoring	[94]
HGSC	PDX	NSG mice	Serum	Protein	N-glycopeptide enrichment	Shotgun proteomics	Potential biomarkers	[95]
PDAC	PDO	N/A	Supernatants	Protein	Ultrafiltration and size exclusion chromatography	LC–MS/MS	Potential biomarkers	[96]
BC	PDO	N/A	Culture media	miRNA	miRNeasy Serum/Plasma mini kit (Qiagen)	qRT-PCR	Drug testing	[97]
CRC	PDO	N/A	Supernatants	EV	Centrifugation and ultracentrifugation	Anti-CD81-coated beads	Mechanism of tumorigenesis	[98]
PDAC	PDO	N/A	Serum-free conditioned media	EV	Centrifugation and ultracentrifugation	Anti-CD63 or anti-CD81-coated beads	Potential biomarkers of high cell proliferation rate	[99]
CRA	PDO	N/A	Conditioned media	miRNA	Ultracentrifugation for EVs miRNeasy Mini Kit for miRNAs	Microarray analysis; qRT-PCR	Diagnostic biomarkers	[100]
PDAC	PDX PXO	Foxn1/Nu	Serum-free conditioned media	protein	Total Exosome Isolation Reagent	LC–MS/MS	Potential screening biomarkers	[101]
CRC	PDO	N/A	Conditioned media	EV	Centrifugation and ultracentrifugation	Anti-CD63 or anti-CD81-coated beads	Potential biomarkers of high cell proliferation rate	[102]

EV, extracellular vesicles; miRNA, microRNA; HCC, hepatocellular cancer carcinoma; SCLC, small cell lung cancer; AML, acute myeloid leukemia; DLBCL, diffuse large B-cell lymphoma; PDAC, pancreatic ductal adenocarcinoma; HGSC, high-grade serous ovarian cancer; CRC, colorectal cancer; CRA, colorectal adenoma; BC, breast cancer; NOD, non-obese diabetic; NSG, NOD scid gamma; SCID, severe combined immunodeficient; N/A, not available; PXO, PDX-derived organoid; qRT-PCR, quantitative real time PCR; LC–MS, liquid chromatography-mass spectrometry; MS, mass spectrometry

The heterogeneity of EVs contents was studied in PDOs. Zeöld et al. found that there’s a certain heterogeneity of miRNAs of EVs from different PDAC organoid-derived models [103]. Importantly, the miRNA profile in the EVs of the model and the matched patient overlapped [103]. In another study, the protein biomarkers identified with cell culture and PDO models had little overlaps [96]. This can be attributed to the dynamic protein profile with the tumor progression, which can be identified in PDO models but not in cell lines.

To isolate tumor-specific EVs, novel analytical techniques based on density, size, immunoaffinity, charge interaction and microfluidics, filed-flow fraction are emerging [104, 105]. Even so, the results of analysis using different methods may be different or even contradictory. And this is the reason why the standard protocols need further optimization [106]. Newly emerging techniques made the patient-derived models more useful. The application of fluorochromes, including indocyanine green (ICG), fluorescein isothiocyanate (FITC), and tetramethylrhodamine-5-isothiocyanate (TRITC), made the route of EVs transport visible in patient-derived models [107, 108]. And multi-organ-on-a-chip (MOC) models combined organoids with microfluidics and expanded the advantages of organoids [109]. They allowed EVs to travel between two or more organs to study the pre-metastasis niche. The emergence of novel techniques will facilitate research on EVs in multiple preclinical models.

Simulating a therapeutic response in patient-derived models is important for testing therapeutic options and pharmacogenomics during drug development. For tissue biopsies with insufficient amounts of material, patient-derived models which expand material for subsequent molecular profiling and functional downstream applications are more than valuable. These models provide a fast platform that allows molecular characterization of patients within a reasonable time [84]. Patient-derived models help identify tumor-specific cfDNA/ctDNA and EVs, providing supplementary information to the clinic [54, 101, 110]. However, there are limitations to studying cfDNA and EVs in patient-derived models. The PDX and PDO models are established from tissue biopsies. In that case, the cfDNA/ctDNA and EVs mainly function in relapse detection and adjuvant therapy outcome evaluation. CDX models seem better for studies of unreachable or early-stage tumors. In addition, we still need prospective studies to clarify the origin of cfDNA [78], the regulation of the fragmentation pattern of cfDNA [111], and the biological role of cfDNA [112]. To exert the clinical potential of EVs, we should also take advantage of patient-derived models and classify EVs into more specific subsets using multi-omics data.

## Conclusion

In this review, we summarized the application of patient-derived models, especially the CDX models, in liquid biopsy research. Patient-derived models are promising preclinical models to study the biology and clinical implementation of CTCs, cfDNA/ctDNA, and EVs. Further, combined with novel multi-omics tools, a patient-derived model will provide valuable answers to important clinical questions.

## Data Availability

Not applicable.

## Abbreviations

AdSCs: Adult stem cells
AML: Acute myeloid leukemia
BC: Breast cancer
BCC: Binary colloidal crystal
CAN: Copy number aberration
CDX: Circulating tumor cell-derived xenograft
cfDNA: Cell-free DNA
CNVs: Copy-number variation
CRA: Colorectal adenoma
CRC: Colorectal cancer
CRPC: Castration-resistant prostate cancer
CSC: Cancer stem cell
CTC: Circulating tumor cell
CTCL: Cutaneous T-cell lymphoma
ctDNA: Circulating tumor DNA
ddPCR: Droplet digital PCR
DDR: DNA damage response
DLA: Diagnostic leukapheresis
DLBCL: Diffuse large B-cell lymphoma
DMEM: Dulbecco's modified eagle medium
dPCR: Digital PCR
DR: Detection rate
EGFR: Epidermal growth factor receptor
EMP: Epithelial-mesenchymal-plasticity
EMT: Epithelial-mesenchymal transition
EpCAM: Epithelial cell adhesion molecule
EVs: Extracellular vesicles
FACS: Fluorescence-activated cell sorting
FITC: Fluorescein isothiocyanate
GB: Glioblastoma
HCC: Hepatocellular cancer carcinoma
HGSC: High-grade serous ovarian cancer
HNC: Head and neck cancer
HSP: Heat shock protein
ICG: Indocyanine green
iPSCs: Pluripotent stem cells
ITH: Intra-tumor heterogeneity
LC-MS: Liquid chromatography-mass spectrometry
MFS: Metastasis-free survival
MOC: Multi-organ-on-a-chip
N/A: Not available
NE: Neuroendocrine
NGS: Next-generation sequencing
NOD: Non-obese diabetic
NSCLC: Non-small cell lung cancer
NSG: NOD scid gamma
OS: Overall survival
PBMC: Peripheral blood mononuclear cell
PCa: Prostate cancer
PCa: Prostate cancer
PCR: Polymerase chain reaction
PDAC: Pancreatic ductal adenocarcinoma
PDO: Patient-derived organoid
PDX: Patient-derived xenograft
pRCC: Papillary renal cell carcinoma
PXO: PDX-derived organoid
qPCR: Quantitative PCR
qRT-PCR: Quantitative real time polymerase chain reaction
RPPA: Reverse-phase protein array
SCID: Severe combined immunodeficient
SCLC: Small cell lung cancer
TNBC: Triple-negative breast cancer
TRITC: Tetramethylrhodamine-5-isothiocyanate
VM: Vascular mimicry
WGS: Whole genome sequencing
